# Arteritic Anterior Ischemic Optic Neuropathy (AAION) Associated with COVID-19 Infection: A Case Report and Review of the Literature

**DOI:** 10.1155/2023/9009925

**Published:** 2023-07-17

**Authors:** Kourosh Shahraki, Amin Najafi, Negin Ashoori, Nayyereh Razzaghpour, Kianoush Shahraki

**Affiliations:** ^1^Department of Ophthalmology, Alzahra Eye Hospital, Zahedan University of Medical Sciences, Zahedan, Iran; ^2^Department of Surgery, School of Medicine and Allied Medical Sciences, Imam Reza Hospital, Ardabil University of Medical Sciences, Ardabil, Iran

## Abstract

Anterior ischemic optic neuropathy (AION) is the most frequent cause of acute optic nerve damage in the elderly, usually causing acute, unilateral, and painless permanent visual loss. Arteritic anterior ischemic optic neuropathy (AAION) is a result of endothelial cell inflammation and the subsequent thrombosis and occlusion in the blood-supplying arteries of the optic nerve head. AAION accounts only for 5-10% of all AION cases that are associated with vasculitis which usually takes place in the course of a giant cell arteritis (GCA). In this paper, we report a case of AAION following a COVID-19 respiratory infection. Although it is uncertain whether SARS-CoV-2 infection triggered the AAION or was coincidental, the possible association of the events is concerning.

## 1. Introduction

Anterior ischemic optic neuropathy (AION) occurs due to optic nerve ischemia. It is the most frequent cause of acute optic nerve damage in the elderly, usually causing acute, unilateral, and painless permanent visual loss. AION is divided into two types with different histopathology and clinical features: arteritic and nonarteritic. Arteritic anterior ischemic optic neuropathy (AAION) is a result of endothelial cell inflammation and the subsequent thrombosis and occlusion in the blood-supplying arteries of the optic nerve head. AAION accounts only for 5-10% of all AION cases [1]. AAION is associated with vasculitis and usually takes place in the course of a giant cell arteritis (GCA) and occurs in patients older than 50 years (70-year-old average) [2]. The nonarteritic type (NAION) which develops in the setting of arteriosclerosis, diabetes, hypertension, hyperlipidemia, etc. occurs in a relatively younger age group (60-year-old average) and is the most common form of AION in the population [2, 3].

AION often presents as an acute painless monocular visual loss with a decreased visual field. Altitudinal visual field defects are common complaints of patients. The diagnosis is made based on clinical features, laboratory results, and ocular examination. A pale optic disc swelling is a diagnostic hallmark for AAION [3]. AAION is an ocular emergency, and treatment must begin immediately to prevent further visual loss in the normal unaffected eye. Treatment includes a high dose of intravenous corticosteroids (methylprednisolone pulse therapy) for 3-5 days followed by oral prednisolone tapered slowly over 12 months or more [4].

In 2019, a highly pathogenic and transmittable virus known as SARS-CoV-2 emerged and caused an infection (COVID-19), which resulted in a worldwide pandemic. Although the most characteristic of COVID-19 infection is an acute respiratory tract syndrome with fever, it can affect almost every organ in the body [5–7]. Ocular manifestations of COVID-19 that are reported till now are various and can involve different structures of the eye from the anterior segment to retinal layers and vasculature and can occur at any time during the disease's progression, but the incidence is generally low [7, 8]. The most typical ocular manifestations are conjunctival injection, conjunctivitis, and ocular surface complaints.

In this case report, we present a 34-year-old female patient with a clear past medical history who developed AAION shortly after being infected with SARS-CoV-2. According to our web search, there was only one reported case of AAION following COVID-19 infection, and this is the second reported case of AAION in COVID-19-infected patients.

Even though it can be an accidental coincidence of AAION with the COVID-19 infection, because of its effect on vascular endothelium, we believe that COVID-19 infection may be considered a risk factor for ocular ischemic events such as AAION.

## 2. Case Report

In the fall of 2021, a 34-year-old woman presented to our retina clinic two days ago with a chief complaint of sudden onset, painless vision loss in her left eye 2 days ago after waking up in the morning. She complained about blurred vision with a scotoma at the inferior eye field. Five days before the onset of the ocular symptoms, the patient was diagnosed with a novel coronavirus infection (COVID-19) based on clinical features such as fever, myalgia, dry cough, dyspnea, and a positive PCR test of nasopharyngeal sample for COVID-19.

She had not been hospitalized because of her stable vital signs and had been under outpatient supportive care at her home. The patient had not done her COVID-19 vaccination, and the history of any previous ocular or systemic diseases was negative. The history of drug history was negative. She did not have a history of significant headaches, scalp tenderness, jaw claudication, weight loss, vomiting, double vision, or any other rheumatologic disease symptoms.

**Figure 1:**
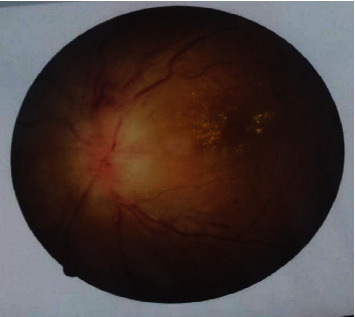
Fundus of the left eye: pale optic disc swelling.

**Figure 2:**
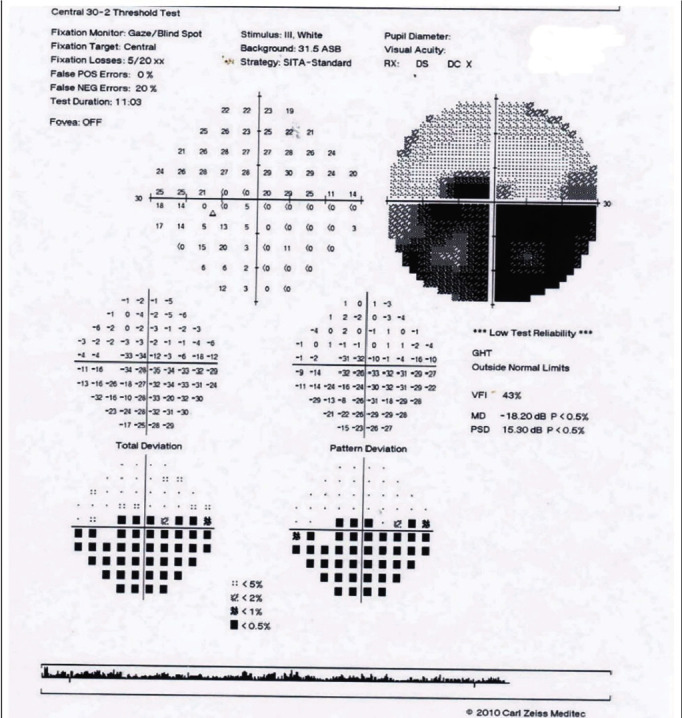
Visual field test of the left eye.

On examination, the best-corrected visual acuity (BCVA) was 1/10 in the left eye (OS) and 10/10 in the right eye (OD). Extraocular motility was in the normal range and painless. Relative afferent papillary defect (RAPD) was grade II positive in the left eye, and red/green color vision according to the Ishihara chart was impaired. On the examination by slit lamp, anterior segment was normal. IOP was 17 mmHg in both eyes. In fundoscopy, the right eye was completely normal, but a pale optic disc swelling, peripapillary hemorrhages, and adjacent macular cotton-wool exudate with edema were observed in the left eye as shown in Figure 1. The confrontational visual field demonstrated an inferior hemifield defect in her left eye. There was an inferior left visual field defect in the perimetry test, with no visual field abnormalities in the contralateral eye (Figure 2). All the blood tests such as the venereal diseases research laboratory (VDRL), human immunodeficiency virus (HIV) antibody, hematological, rheumatological, and immunological tests were normal but high erythrocyte sedimentation rate (ESR) and *C-reactive protein* (CRP).

Based on the clinical features (unilateral painless visual decrease), laboratory tests (elevated levels of ESR and CRP), and ocular examination (pale optic disc swelling in fundoscopy), a diagnosis of AAION was considered. The patient was admitted to the hospital, and treatment with a high dose of intravenous methylprednisolone (1 g/day) for three days was started. In laboratory tests, an elevated C-reactive protein (CRP) (3+) and erythrocyte sedimentation rate (ESR) (32 mm/h) with a decreased level of vitamin D3 (14 ng/ml) were detected. The results of the complete blood test, coagulopathy tests (PT, PTT, INR, protein C, protein S, and antithrombin), and rheumatologic tests (RF, ANA, and ANCA) were within the normal range. Brain and orbital MRI of the patient was normal; rheumatologic consultation also was done that suggested following up with the patient. After 3 days, the patient was discharged with oral prednisolone (1 mg/kg/day).

After one week at the follow-up visit, the patient's visual acuity of the left eye was 2/10, and disc edema was resolved. Finally, after four months of follow-up, the patient's left eye visual acuity improved to 3/10, fundoscopic examination revealed the resolution of left optic disc edema and optic nerve head paleness with restricted visual field, while the right eye was fortunately normal and not affected by the disease.

## 3. Discussion

The AAION results from the optic disc infarction due to inflammation and the subsequent thrombosis and occlusion usually in the short posterior ciliary arteries (SPCA), but the specific location of the microvasculopathy is still unclear. The optic nerve head blood supply is driven from SPCA branches that originate from the internal carotid artery.

Most often, AAIONs are associated with giant cell arteritis (GCA) vasculitis and SPCA involvement. Rarely, it can also result from other types of vasculitis, such as systemic lupus erythematosus, polyarteritis nodosa, and herpes zoster [4].

In this report, a diagnosis of AAION was made based on the clinical presentations, laboratory tests, and examination of a patient with a recent history of COVID-19 infection. The diagnosis of GCA seems unlikely based on the patient's age and the absence of GCA symptoms. Rheumatologic consultation ruled out any other possible causes.

The vascular endothelium is affected by both AAION and COVID-19. We believe that the ability of the SARS-CoV-2 virus to invade the vascular endothelium provides an important link between AAION and COVID-19 infection. The virus can directly infect the vascular endothelial cells through endocytosis by binding the spike protein to the host cell receptor angiotensin-converting enzyme 2 (ACE2), which is present on both the vascular endothelial surface and the respiratory membrane. This invasion can lead to vasculitis, endothelialitis, and microvascular damage and thus may compromise ocular circulation [5, 9–11].

Since the mechanisms implicated in antigen/receptor interaction are of key importance for cell infection, several recent review articles refer to SARS-CoV-2 binding to ACE2, which may increase tissue susceptibility to COVID-19 infection [12, 13]. These receptors are found on respiratory epithelial cells as well as other tissues such as vascular endothelial cells, smooth muscle cells in arteries, immune cells, and even neurons [14–16].

Zhou et al. in their study showed that the presence of ACE2 in the retinal endothelium causes the potential susceptibility of the retina to SARS-CoV-2 infection. Neuroretinal infection and damage can occur as a result of direct infection and destruction of endothelial cells, as well as systemic inflammation [17].

There are some predisposing factors in COVID-19 patients such as hypoxia and respiratory distress, prone positioning, increased venous pressure, and IOP that may damage ocular perfusion pressure which may cause AAION.

Vasculitis has been identified as a COVID-19 complication caused by epitheliopathy, and numerous studies have found an increased incidence of vasculitis following SARS-CoV-2 infection [9]. Roncati et al. stated that an escalation from type 2 T-helper immune response to type 3 hypersensitivity occurs in COVID-19-induced vasculitis [18].

On the other hand, several hypotheses have been reported that explain the effect of the inflammatory response following SARS-CoV-2 infection, described as a “cytokine storm.” This process can cause thrombin generation and reduced fibrinolysis, which activates the coagulation cascade and hypercoagulable state. COVID-19-associated coagulopathy (CAC) can cause various thromboembolic complications with a pathogenesis that may cause endothelial injury and an increase in circulating prothrombotic factors [19, 20]. Pisano et al. reported a case with large vessel occlusion secondary to COVID-19 hypercoagulability with high D-dimer [21]. Chan et al. reported an ischemic colitis case with high D-dimer levels and inflammatory markers that highlighted the possible hypercoagulable state in patients with SARS-CoV-2 infection [22].

In general, the three mechanisms listed below cause a hypercoagulable state and occlusion of blood supply arteries:

1. An increased level of clotting factors (VWF, factor VIII, and fibrinogen) is released by the stimulated endothelial cell
2. ACE2 activity loss causes increased angiotensin II levels and vasoconstriction
3. Suppressed production of nitric oxide (NO) causes leukocyte and platelet adhesion and vascular constriction [10]

The combination of all the mentioned mechanisms described above can lead to conditions that can trigger a sudden onset of AAION. According to our search, few published reports of the coexistence of COVID-19 and AAION are rare. Similarly, Szydełko et al. reported a patient who developed GCA with ophthalmic manifestations of AAION shortly after COVID-19 infection [23]. In a retrospective study, Luther et al. observed a significant increase in the number of patients diagnosed with GCA and GCA-related visual impairment during the postpeak period of the COVID-19 pandemic in the United Kingdom [24].

In contrast, there are more case reports on NAION following COVID-19. Golabchi et al. and Babazadeh et al. described two separate cases diagnosed with NAION in the setting of COVID-19 [25, 26]. Rho et al. reported a case of NAION in a patient with COVID-19 that occurs following endothelial cell injury by hypercoagulability and low oxygen state [27]. Thus, beyond the fact that it seems NAION is more prevalent in COVID-19 infection, but AAION may also be seen after COVID-19 infection as a result of direct SPCA endothelial cell damage by a virus.

## 4. Conclusion

Patients with COVID-19 are prone to thromboembolic and ischemic events, and it can appear as a potential risk factor for ocular ischemic events such as AAION. Therefore, AAION can be considered another ocular manifestation of COVID-19. Accordingly, physicians should attentively evaluate COVID-19 patients with new-onset visual complaints as it can be an ocular emergency. However, further studies are needed to investigate the underlying pathogenesis of COVID-19 and its association with AAION.

## Data Availability

The data used to support the findings of this study are available from the corresponding author upon request.
